# Intelligent and Energy-Autonomous Wearable and Implantable Biosensors: Nanomaterial Interfaces, Energy Harvesting, Edge AI, and Long-Term Reliability

**DOI:** 10.3390/bioengineering13101129

**Published:** 2026-09-27

**Authors:** Stefano Bellucci

**Affiliations:** 1 National Institute of Materials Physics, Atomistilor 405A, 077125 Măgurele, Romania; stefano.bellucci@infim.ro; 2 Qi S.r.l., Via Monte d’Oro 2/A, 00071 Pomezia, RM, Italy; 3 Universidad Ecotec, Km 13.5 Samborondón, Samborondón 092302, Ecuador

**Keywords:** wearable biosensors, implantable biosensors, nanomaterials, self-powered sensing, triboelectric nanogenerator, wireless power transfer, edge AI, neuromorphic computing, bioelectronics, reliability

## Abstract

Wearable and implantable biosensors are becoming small distributed biomedical systems rather than isolated transducers. Their practical performance depends on how the sensing interface, analog front end, power source, local computation, wireless link, packaging, and therapeutic output interact over time. The analysis focuses on that cross-layer problem, with emphasis on nanomaterial interfaces, energy autonomy, edge intelligence, and long-term reliability. Graphene, carbon nanotubes, MXenes, and transition-metal dichalcogenides are discussed across electrochemical, field-effect, impedance, optical, and radio-frequency transduction. Mechanical nanogenerators, biofuel cells, wireless power transfer, and hybrid storage are compared using the energy actually available after rectification and regulation rather than peak generator output alone. Quantitative re-plots illustrate non-monotonic carbon-nanotube loading in triboelectric layers and voltage-tunable few-layer-graphene microwave components. Edge AI is treated as part of the power and measurement architecture: local inference can reduce radio traffic and latency, but introduces model drift, uncertainty, and update requirements. The final sections connect biofouling, encapsulation, mechanical fatigue, calibration drift, wireless safety, and algorithm lifecycle to a common validation ladder for wearable, insertable, and implantable systems.

## 1. Introduction

Wearable and implantable biosensors now occupy a space between analytical chemistry, soft electronics and medical-device engineering. [1] A skin patch that measures metabolites, an insertable sensor interrogated by a wearable reader, and a neural implant that records and stimulates tissue are very different products, yet they share the same systems problem: a useful biological signal must cross a material interface, survive analog conditioning, be interpreted with a finite energy budget, and remain trustworthy during motion, hydration, aging and repeated use. Recent reviews have described sensing mechanisms and closed-loop therapeutic applications [2] and have surveyed non-invasive wearable biosensing in body fluids and multimodal devices [3]. Those perspectives are essential, but they leave a second question open: how should the sensing material, power architecture, computation and communication be co-designed when the device must operate continuously on or inside the body?

Wireless power and data transfer have consequently become inseparable from materials and mechanical design in flexible bioelectronics [4]. Energy autonomy is also broader than eliminating a battery. Piezoelectric and triboelectric nanogenerators can transform body motion into electrical signals and harvested energy, while the same mechanical coupling can be used directly for self-powered sensing [5]. Implantable versions extend this idea to heartbeat, respiration, muscle deformation and externally delivered ultrasound, but their available energy must be reconciled with encapsulation, tissue motion and therapeutic loads [6]. At the opposite end of the power budget, moving part of the computation to the sensor node reduces continuous radio transmission and can improve latency and privacy. Systematic mapping of wearable edge-computing literature already shows a rapid convergence between machine learning and local biomedical sensing [7].

The energy cost of intelligence is therefore becoming a design variable. Tiny machine learning (TinyML) has been developed specifically for resource-constrained cardiovascular and wearable platforms, using quantization, pruning, knowledge distillation and efficient network architectures to reduce memory and computation [8]. On-device learning and micro-training approaches go a step further by updating models locally, which is attractive for personalized physiological signals but difficult to verify safely when the sensor itself drifts [9]. An important hardware endpoint appeared in 2025 with an all-printed chip-less wearable neuromorphic system that integrates multimodal physicochemical sensing with on-body processing [10]. Emerging resistive, ferroelectric and photonic memories are being explored for the same reason: in-memory and event-driven computation can reduce data movement in the Internet of Medical Things [11]. Memristive biomedical systems now span sensing, neural processing and therapeutic integration, although device variability and chronic biocompatibility remain open constraints [12].

This review treats intelligent wearable and implantable biosensors as cross-layer systems. It starts at nanomaterial-enabled interfaces, moves through energy harvesting and power delivery, and then examines front-end circuits, RF telemetry, edge AI and closed-loop therapy. The final criterion is not the largest sensitivity, harvested voltage or classification accuracy considered separately. It is stable information per unit energy over the intended lifetime, offering enough evidence to explain when the device is outside its validated operating domain.

## 2. Scope and Critical Review Strategy

This work was conducted as a structured critical narrative review rather than a systematic review or meta-analysis. Targeted literature searches were updated through 22 September 2026 using PubMed and journal/publisher search platforms, including Nature Portfolio, ACS Publications, RSC Publishing, IOP Publishing, Springer Nature, MDPI, and Elsevier/ScienceDirect, supplemented by backward and forward citation tracing from relevant reviews and primary studies. Search strings combined device terms (wearable, implantable, insertable, biosensor, bioelectronics), materials terms (graphene, carbon nanotube, MXene, transition-metal dichalcogenide, conductive polymer), power terms (triboelectric, piezoelectric, biofuel cell, wireless power, energy harvesting), intelligence terms (edge AI, TinyML, neuromorphic, machine learning, out-of-distribution), and reliability/translation terms (biofouling, encapsulation, drift, fatigue, sterilization, calibration, closed loop, in vivo). Searches prioritized 2020–2026 publications; older studies were retained only when they established a device principle, benchmark method, or controlled quantitative series used in the present analysis. Records were screened first for direct relevance to the cross-layer scope and then at full-text/abstract level when quantitative device information was required. Included studies had to report a clearly identifiable sensing, power, communication, computation, therapeutic, or reliability function relevant to wearable/implantable systems. Purely conceptual articles without device-level evidence, duplicate reports, and studies lacking sufficient methodological detail for the claim being discussed were excluded from quantitative comparisons. Review articles were used to map the field and identify primary studies, whereas quantitative tables preferentially used original reports. Evidence was assigned to four classes according to the highest demonstrated level: E1, material/component evidence; E2, integrated wearable/implantable prototype; E3, realistic-matrix, on-body, or in vivo demonstration; and E4, longitudinal or clinically relevant operation. When a study supported more than one class, the highest experimentally demonstrated class was reported, while untested downstream claims were not promoted to a higher level. Because reporting conventions differ strongly across transduction, power, AI, and implant studies, missing quantities are reported as NR (not reported) rather than inferred.

Quantitative extraction followed a conservative rule: values were transcribed from the primary article or supplementary information, normalized only when the denominator and operating condition were explicit, and otherwise retained in the authors’ native units. Cross-platform comparisons identify engineering ranges and reporting gaps rather than rank heterogeneous devices.

Figure 1 anchors the cross-layer discussion in experimentally implemented systems. The three reference architectures span a fully integrated microneedle array for continuous multi-analyte monitoring in volunteers [13], a wireless microneedle platform for longitudinal nucleic-acid monitoring [14], and a wireless battery-less implant for multimodal closed-loop neuromodulation [15]. Although their analytes and therapeutic functions differ, each makes the same system-level coupling visible: the biointerface, analog front end, power/data path, computation or control, and validation procedure must be evaluated together.

Table 1 summarizes the different design envelopes of wearable and implantable systems. The division is not absolute: insertable sensors deliberately separate the implanted sensing material from the wearable electronics, and many closed-loop platforms distribute computation and power between the body and an external unit. Nevertheless, the distinction helps prevent inappropriate benchmarks. Milliwatts that are acceptable in a rechargeable wrist module can be excessive inside tissue, while an implant can sometimes tolerate a mechanically stable rigid package that would be uncomfortable on skin.

Figure 2 makes this contrast explicit. Wearables have greater physical area and easier maintenance but operate in a highly variable mechanical and chemical environment. Implants can be sheltered from external abrasion yet face a much stricter thermal, biological and maintenance budget. In both cases, the best architecture is the one that preserves calibrated information under the dominant real-world disturbance rather than the one that maximizes an isolated laboratory metric.

## 3. Nanomaterial Interfaces and Transduction

### 3.1. Why Nanomaterials Matter at the Biointerface

Nanomaterials can improve a biosensor for several physically distinct reasons: more electrochemically active area, faster electron transfer, higher carrier mobility, strong optical confinement, tunable surface chemistry, local mechanical compliance, or a large impedance change under adsorption. Those mechanisms should not be collapsed into a generic statement that a material ‘increases sensitivity’. Graphene illustrates the problem well. Reviews of graphene surface-plasmon-resonance biosensors emphasize the combination of large surface area, optical/electrical tunability and adsorbate interaction [16], while electrochemical reviews show that defects, functional groups, nanostructuring and composite formation determine the practical electron-transfer response [17]. Field-effect transistor (FET) biosensors add a further constraint because the transduction is controlled by channel electrostatics, Debye screening, contact resistance and drift in addition to receptor chemistry [18].

Primary device work helps separate these mechanisms. A bottom-up graphene nanoplatelet device showed measurable resistance changes during acetylene exposure from low pressures, establishing that a nanocarbon network can convert molecular adsorption into a resistive signal [19]. Screen-printed carbon platforms can exploit the same electron-transfer logic in a clinically closer form; a uric-acid biosensor based on modified screen-printed electrodes illustrates the value of a manufacturable electrochemical interface rather than an ideal planar material [20]. Related modified electrochemical platforms have been used for sensitive pollutant detection, again showing that surface functionalization and electron transfer determine the analytical response more directly than nominal nanocarbon content [21].

For devices that contact skin or tissue, the useful nanomaterial state is also the state that can be manufactured and maintained. A high electroactive area is of little value if the coating cracks during bending, delaminates during hydration or presents a surface chemistry that changes after sterilization. The practical unit of design is therefore the complete interphase: substrate, conductive network, receptor layer, electrolyte or tissue, and encapsulation edge. Graphene and CNT networks are especially sensitive to this distinction because electrical properties depend strongly on contact between flakes or tubes. Small changes in swelling or strain can alter those contacts and create apparent analyte responses. In a wearable sensor, this can appear as a motion artifact; in an implant, the same mechanism may be mistaken for slow biological drift. A convincing interface study should therefore pair analytical calibration with impedance or resistance monitoring, mechanical cycling, and a description of the hydrated surface state.

Nanomaterial selection also changes the calibration problem. Two-dimensional materials can supply large surface-to-volume ratios and strong field coupling, but they often respond to more than one environmental variable. Ionic strength, pH, temperature, nonspecific adsorption and local hydration may all change carrier density or interfacial capacitance. Selectivity consequently cannot be assigned to the nanomaterial alone. It emerges from receptor chemistry, blocking strategy, transport geometry and signal processing. This is one reason multichannel and differential architectures are valuable: a reference channel that experiences the same strain, temperature and hydration but lacks the specific receptor can be more useful for long-term operation than a further increase in nominal sensitivity.

### 3.2. Impedance, FET, Optical and RF Readout

Impedance spectroscopy is particularly attractive for intelligent low-power systems because one interface can be probed at several frequencies and changes can be separated into resistive, capacitive and interfacial contributions. Electrical-impedance monitoring of graphene nanoplatelet filters demonstrated real-time tracking of material state during exposure to organic pollutants, using the spectral response as a state-of-health observable rather than relying on one dc resistance value [22]. The same principle is transferable to wearable electrodes, hydrogel interfaces and implanted coatings: frequency dependence can distinguish electrode degradation, contact changes and analyte-dependent processes if the analog front end is stable enough.

Radio-frequency and terahertz transduction extend impedance sensing into a resonant domain. Electrochemically gated graphene microwave waveguides have demonstrated broadband, high-sensitivity biosensing in which biochemical interaction changes the guided microwave response [23]. Reviews of graphene terahertz devices place sensing and communication in the same material platform, because conductivity tuning can alter both local field confinement and circuit-level transmission [24]. Optical two-dimensional materials provide another route. Bilayer MoS2 has been modeled in surface-plasmon-resonance architectures for SARS-CoV-2 sensing [25], and WS2/Si3N4 designs illustrate how a dielectric/2D-material stack can be optimized for low-concentration coronavirus detection [26]. At terahertz frequencies, hybrid low-dimensional-material metasurfaces have been proposed for dopamine detection, where resonance displacement is the analytical observable rather than a current or voltage [27]. These optical and THz examples remain mostly device-model studies; they are valuable as interface physics, but should not be described as wearable or implantable systems without integration evidence.

Wireless sensing can also be achieved without an active RF transmitter at the sensor. A graphene-based magnetoelastic biosensor has been demonstrated for wireless COVID-19 antibody detection, using resonance interrogation as the remote readout [28]. 3D-printed hollow microneedle architectures are moving in the opposite direction by integrating electrochemical sensing with wearable electronics and wireless glucose monitoring [29]. These examples represent two useful endpoints: passive or remotely interrogated resonators minimize local electronics, while electrochemical microneedles bring a rich front end close to the biological sample. The appropriate choice depends on penetration depth, analyte transport, required sampling rate and the available power budget.

Figure 3 compares three concrete sampling arrangements from primary studies. The examples distinguish direct microneedle access to interstitial fluid [13], reverse-iontophoretic enrichment followed by microneedle electrochemical sensing [14], and a sensing-controlled hollow-microneedle insulin-delivery loop [30]. This comparison makes sampling time, biological compartment, signal conditioning, and downstream action explicit.

Integrated wearable systems increasingly combine these interfaces with software. A smartphone-linked sweat glucose device coupled wearable electrochemistry with machine learning analysis for real-time diabetes screening [31]. A separate wearable platform used explainable features from a non-enzymatic sweat sensor to predict tyrosine, tryptophan and pH during exercise [32]. Self-powered biochemical systems are beginning to add the same computational layer: a bipolar self-powered sensor integrated multivariate miRNA measurement with smartphone readout and machine learning prediction [33]. These studies are important because they make the signal-processing burden visible. Selectivity is no longer produced only by receptor chemistry; it can emerge from the combination of partially selective channels, temporal features and a trained model.

Component-level pollutant, optical/THz, and microwave examples in Section 3.2 and Section 5.2 are retained only as enabling evidence. They establish transfer functions, materials physics, or circuit tunability that may be useful in biomedical systems, but they do not constitute wearable or implantable validation. Biomedical relevance requires physiological-matrix operation, mechanical compliance, tissue/skin loading, packaging, power-budget, safety, and longitudinal-calibration evidence [13,34,35,36,37].

## 4. Energy Autonomy: Harvesting, Storage and Wireless Power

### 4.1. Self-Powered Does Not Mean Power-Free

Triboelectric nanogenerators (TENGs) convert contact electrification and electrostatic induction into alternating electrical output [38]. Their reported voltage can be large even when the available average power is small, so material and device comparisons require a controlled definition of transferred charge, load, active area and operating condition. Figure-of-merit work on TENGs established surface charge density and structural normalization as a more useful basis than peak open-circuit voltage alone [39]. This distinction becomes critical in biomedical systems. A TENG used directly as a motion sensor may need almost no stored energy; a TENG that is expected to run an ADC, microcontroller and radio must be evaluated after rectification, regulation and storage losses [40].

### 4.2. Nanocomposite TENGs: Controlled Evidence for a Useful Filler Window

Controlled nanocomposite series show why ‘more conductive filler’ is a poor optimization rule. In polysiloxane/MWCNT films, open-circuit voltage increased from 32 V for the pristine polymer to 51 V at 0.05 wt% MWCNT, and then fell to 37 V at 0.10 wt%; the corresponding power-density series rose from 33.0 to 90.8 mW m^−2^ and then fell to 46.3 mW m^−2^ [41]. Introducing controlled porosity into the same material family provided a second optimization axis: reported power density increased from 122.5 mW m^−2^ for the dense film to 280.6 mW m^−2^ at an intermediate pore size, and then decreased for the largest-pore structure [42]. Nylon/MWCNT devices show a similar concentration optimum, with open-circuit voltage increasing from 17.5 V for pristine nylon to 29.7 V at 0.05 wt% and declining at 0.10 wt% [43].

Figure 4 re-plots the concentration series on a common normalized basis. What matters in this comparison is the shape of the response, not an absolute cross-device ranking. In both polymer matrices, the voltage reaches a maximum at low MWCNT loading and declines as the composite approaches a more lossy conductive state. This is consistent with a competition between interfacial polarization/charge trapping and aggregation, tunneling and dielectric leakage. The implication for wearables is direct: a nanofiller that raises peak electrical output may also stiffen the interface, increase drift or reduce charge retention. The useful composition is a system window rather than a material maximum.

Other low-dimensional networks illustrate different trade-offs. CNT/Nb2CTx/PEDOT hybrid films combine one-dimensional and two-dimensional conduction paths with a conductive polymer and have been used in flexible self-powered wearable sensing [44]. MXene/TPU films emphasize extreme stretchability and mechanical compatibility [45], whereas MXene/PVA hydrogel TENGs couple deformability with ionic/electronic conduction and water-dependent mechanics [46]. A graphene-oxide-coated silk-fibroin/PCL fibrous TENG is notable because biomedical relevance was considered together with operation during prolonged buffer exposure [47]. These examples show why energy harvesting cannot be optimized separately from the mechanical and chemical environment of the body.

Biomedical excitation is itself variable. Gait, respiration and pulse produce different force spectra from the calibrated mechanical shakers commonly used to characterize nanogenerators. Mounting pressure, garment fit, skin curvature and soft-tissue damping further change the amplitude delivered to the active layer. For this reason, the laboratory optimum in filler concentration or porosity should be treated as a starting composition rather than a fixed wearable optimum. The same sample should be tested under the intended strain amplitude and frequency distribution, including low-amplitude events that dominate ordinary daily use. Reporting only the maximum output under vigorous excitation overstates the energy available to an always-on sensing node and gives little information about signal fidelity in normal activity.

The distinction between harvesting and direct self-powered sensing is equally important. When the triboelectric waveform itself encodes pressure, gait or joint motion, the relevant quantities are sensitivity, linearity, hysteresis, repeatability and waveform stability. When harvested energy is routed to another biochemical sensor, the relevant quantities become rectified energy per event, storage leakage, cold-start behavior and the minimum interval between measurements. A device can be an excellent direct sensor and a poor power supply, or the reverse. Keeping these two architectures separate avoids using a high open-circuit voltage as evidence for energy autonomy.

For Figure 4, numerical values were extracted from the concentration series in Refs. [41,43]. Open-circuit voltage was normalized within each material series to its own pristine-polymer value; power density was retained in native units and was not normalized across laboratories. No interpolation, smoothing, or uncertainty pooling was applied. Because matrix, geometry, excitation and loading differ, the re-plot supports only a within-platform intermediate-loading optimum, not a universal MWCNT concentration or cross-platform ranking.

### 4.3. Wireless Power, Biofuel Cells and Hybrid Storage

Implantable devices often cannot harvest enough physiological energy continuously, so wireless power transfer (WPT) remains a central architecture. Inductive, resonant, capacitive, acoustic and optical methods have different receiver-size, depth, alignment and tissue-heating constraints [48]. More recent comparisons include electromagnetic, magnetoelectric, acoustic and magneto-dynamic modalities and show that no one method dominates across all implant locations [49]. Electromagnetic systems can provide relatively high power but are sensitive to alignment and absorption; acoustic links are attractive at depth but depend on acoustic coupling; magnetoelectric approaches reduce some RF-loss constraints but introduce material and packaging challenges.

Biochemical energy harvesting offers a different route for low-power sensing. Enzymatic biofuel cells can use glucose, lactate and other endogenous fuels as both energy sources and analytical targets, but enzyme lifetime, electron transfer and power density remain practical limits [50]. Hybrid self-powered biosensing architectures combine biofuel cells with capacitive storage or nanogenerators to improve signal intensity and bridge intermittent loads [51]. These systems make clear that an energy-autonomous biomedical node is usually a power-management problem rather than a single-harvester problem: the realistic architecture may contain an energy harvester, a small battery or supercapacitor, a rectifier, and a regulator, and may also display aggressive duty cycling.

Energy budgeting should start from the load rather than from the harvester. The analog front end may require a low continuous baseline, the processor may require a short burst during inference, the radio may require a larger burst during connection or data transfer, and a therapeutic actuator may require a still larger but infrequent reserve. The power-management circuit must satisfy all four needs without allowing storage voltage to fall into a regime where measurement accuracy changes. Consequently, energy-neutral operation should be defined over a stated time window and physiological activity profile. A device that balances its daily energy budget during walking but depletes its storage during sleep is not autonomous for continuous monitoring. The same reasoning applies to WPT: delivered dc power and temperature rises at the intended depth are more informative than coupling efficiency measured between unloaded coils.

Table 2 compares the main energy options from the perspective of an intelligent biosensor rather than from that of an energy harvester alone.

## 5. Analog Front Ends, RF Tunability and Biotelemetry

### 5.1. The Front End Sets the Usable Information Bandwidth

A biosensor interface is not useful until its output is conditioned within the noise and dynamic range of the electronics. Electrochemical currents may require transimpedance amplification; impedance sensors require stable excitation and coherent demodulation; neural interfaces require high-input-impedance low-noise amplifiers; resonant sensors require phase- and amplitude-stable interrogation. These circuits often dominate the baseline power budget because they remain active while the radio and processor sleep. Accordingly, reducing electrode impedance, narrowing the required bandwidth or extracting a richer spectral feature can save energy downstream.

Front-end design should begin with the physiological bandwidth and the expected signal amplitude. Oversampling an intrinsically slow metabolite concentration wastes conversion, memory and radio energy; undersampling a neural or cardiac event can remove the temporal structure that an edge model needs. Input-referred noise should be reported over the actual bandwidth rather than as a single spectral-density value, and ADC resolution should be interpreted together with gain and sensor drift. For impedance or resonant readout, excitation amplitude is also part of the biology: excessive voltage can polarize an electrochemical interface, while excessive RF power can create heating or alter the very medium being measured. These limits should be decided before selecting the machine learning model because they determine the information that reaches it.

Reference and self-test paths are particularly valuable in long-lived devices. A known calibration impedance, dummy electrode, optical reference, or internally generated test waveform can identify front-end gain changes without requiring an external biological standard. This creates a separation between sensor aging and electronics aging that is otherwise difficult to recover from the measured output. It also gives edge software an interpretable sensor-health variable. Rather than asking a classifier to learn every form of hardware drift, the system can explicitly flag a failed reference check and suspend clinical inference.

### 5.2. Few-Layer Graphene as a Tunable RF Material

Few-layer graphene (FLG) microwave components provide a materials-to-circuit foundation for this discussion. A broadband microstrip attenuator demonstrated that electrically controlled graphene resistance can tune microwave transmission over a wide frequency range [52]. An improved microstrip attenuator later achieved an attenuation change from approximately 0.3 to 15 dB at 3 GHz as the bias increased to 6.5 V, corresponding to a large reduction in effective graphene resistance [53]. In a planar patch antenna, voltage-controlled FLG loading shifted the measured resonance from 5.05 GHz at 0 V to 4.50 GHz at 5 V, a 550 MHz change exceeding 10% of the initial frequency [54]. A related phase shifter produced about 40° of tunable phase shift around 5–6 GHz with an insertion-loss penalty of roughly 3 dB [55]. Multi-section input-matched attenuators extended the same principle to substantially larger controllable insertion loss [56].

These components were not wearable or implantable biosensors, and they should not be presented as such. Their relevance is more specific: they demonstrate a large and reproducible transfer function from nanocarbon electrical state to microwave amplitude, phase and resonance. In a biomedical system, the same property can support tunable matching, adaptive antennas, resonant interrogation or impedance-sensitive telemetry if the graphene element can be integrated with a stable biointerface. Figure 5 summarizes the quantitative range of the reported tunability.

The FLG studies are therefore treated as a compact component benchmark. Resonance shift can support adaptive matching under tissue loading; controllable attenuation can extend receiver dynamic range; phase tuning can support field control; and voltage-dependent impedance can provide a reconfiguration variable. None is clinically meaningful until evaluated with tissue-equivalent loading, encapsulation, motion-induced detuning, SAR/temperature rise where applicable, and a complete link budget.

### 5.3. Wireless Data Links Should Be Designed with the Biological Channel

Communication architecture depends strongly on where the electronics reside. BLE is practical for many wearables, NFC can support short-range battery-less interrogation, and backscatter can reduce local radio power when an external reader is available. Implants require a tissue-aware link budget. Recent in-body to on-body intrabody communication measurements show why anatomical path, electrode geometry and frequency cannot be abstracted into an ordinary free-space link [57]. For very small implants, wireless power and data may share the same coil, acoustic link or resonant structure. The communication choice therefore feeds back into encapsulation thickness, antenna size, tissue heating and the energy available to edge computation.

Telemetry should be dimensioned around the information rate rather than the raw sampling rate. A sweat sensor may need only a slowly updated concentration and quality flag; an ECG patch may transmit beats and short exception windows; a neural interface can generate data rates that make continuous raw streaming impractical. Edge compression and event detection therefore have a direct radio-energy value. At the same time, aggressive reduction in transmitted data can make later auditing impossible. A useful compromise is hierarchical communication: low-rate features during normal operation, richer waveform segments around detected events, and full diagnostic streaming during calibration or suspected failure.

For implants, communication performance must also be evaluated after packaging and in a realistic body-equivalent medium. Encapsulation changes effective permittivity and detunes antennas or resonators; tissue composition changes with location and subject; posture can alter the path to an external reader. Link margin should therefore include expected anatomical variation and misalignment rather than a single best-case distance. When power and telemetry share a channel, loss of coupling can simultaneously reduce available energy and data integrity, so the controller needs a defined behavior for that coupled failure.

Table 3 brings together representative systems and component studies. The evidence level column is deliberately explicit: a material or circuit foundation is not equivalent to an on-body or in vivo demonstration.

## 6. Edge AI and Neuromorphic Intelligence

### 6.1. What Should Run on the Body?

Machine learning at the edge is justified when it reduces radio traffic, shortens decision latency, improves privacy or allows the device to adapt to a user-specific baseline. It is not justified simply because a model can be trained. Advanced electrochemical-biosensor reviews increasingly treat ML as part of the analytical pipeline, particularly for multivariate sensor arrays and drift compensation [61]. A striking example outside classical biosensing is robust chemical analysis with graphene chemosensors, where machine learning helps separate overlapping responses and device variability [62]. GFET biochips have similarly combined multiplexed sensing with machine learning to improve parallel discrimination of target antibiotics [63]. These examples support a general principle: data-driven selectivity is useful when the input channels contain complementary physical information and the model is trained across the variability expected at deployment.

Localized surface-plasmon-resonance sensing has also been paired with machine learning for SARS-CoV-2 particle detection [64], and recent work in plasmonic biosensing uses explainable or predictive models to map complex optical features to concentration. The same direction appears in machine learning-enhanced plasmonic sensing more broadly [65]. For wearable and implantable systems, however, the relevant question is not only prediction accuracy. Memory footprint, multiply–accumulate count, wake-up frequency, inference latency and the energy cost of moving data to and from memory determine whether the model belongs on the body.

### 6.2. TinyML, Event-Driven Computation and Model Uncertainty

Figure 6 presents validation workflows from implemented biomedical systems. The examples show three complementary routes to representativeness: human on-body testing against blood/breath reference measurements [13], longitudinal molecular monitoring compared with PCR/RT-PCR [14], and closed-loop glucose sensing/insulin delivery checked against a commercial glucometer and Clarke-error-grid analysis [30]. These workflows illustrate why model or sensor performance is meaningful only when the sampling context and reference method are stated.

Neuromorphic hardware offers a route to lower data-movement cost further. The all-printed chip-less platform already noted [10] combines sensing and local neuromorphic processing in a mechanically conformal device. Emerging-memory approaches aim to perform computation close to storage [11,66], while memristive systems can combine sensing, non-volatile state and adaptive processing [12]. The attraction is strongest for sparse biosignals such as neural spikes, cardiac events or threshold crossings, where event-driven processing avoids a continuously clocked high-rate pipeline. The corresponding verification problem is device variability: an analog synaptic weight that drifts with temperature, humidity or cycling is both a hardware error and a model error.

Dataset construction is as important as model architecture. Repeated measurements from the same sensor, fabrication batch or subject are highly correlated; a random sample-level split can therefore put near-duplicates into both training and test sets. For a wearable intended for new users, evaluation should be subject-wise. For a disposable sensor intended for generalization across manufacturing lots, the test set should contain unseen devices or batches. If personalization is part of the intended workflow, performance should be reported both before and after adaptation, with the amount of calibration data and update energy stated explicitly. This distinction prevents personalization from being confused with generalization.

Explainability is most useful when it can be checked against sensor physics. Feature attribution that identifies temperature, impedance magnitude or a particular spectral region as important should be compared with controlled perturbations of those variables. An explanation is not proof of causality, but disagreement with a well-characterized transduction mechanism can expose leakage or confounding. For embedded systems, simpler models can therefore be preferable even when a larger network improves average accuracy slightly: an interpretable feature set, calibrated uncertainty and a clear abstention rule may produce a safer device with lower memory, verification and energy cost [61,62,63,64,65].

Table 4 summarizes edge-intelligence strategies in terms that are useful for biomedical hardware. The table deliberately includes failure modes because compression or local learning can improve average energy while making worst-case behavior harder to predict.

## 7. From Monitoring to Closed-Loop Therapy

Closed-loop systems raise the engineering threshold because the biosensor becomes part of a controller. Stable tissue interfaces, soft mechanical integration and multimodal acquisition are central to recent closed-loop bioelectronics [69]. Implantable bioelectronic reviews now treat physiological recording and neuromodulation as one feedback architecture spanning neural, cardiovascular, respiratory, digestive, urinary and musculoskeletal targets [70]. In this setting, sensitivity is only one term in the control error. Delay, drift, false events, actuator saturation and communication failure can all change therapy.

A 2025 bimodal epilepsy implant illustrates the integrated endpoint. The system combines an ultraflexible neural probe, embedded LSTM-based seizure detection, immediate electrical stimulation and an on-demand drug capsule. In vivo mouse experiments reported rapid electrical suppression and a slower pharmacological component, explicitly using the two actuators to cover different therapeutic timescales [71]. The importance of this example is architectural: sensing, inference and therapy are coordinated rather than simply packaged together. A similar logic applies to closed-loop drug delivery, cardiac pacing or autonomic neuromodulation. The controller must know not only what the biosensor reports but also whether the sensor is trustworthy at that moment.

Energy autonomy changes again in closed-loop operation. A monitoring-only implant can remain asleep most of the time and transmit occasional summaries. A therapeutic implant must reserve enough energy for the worst credible intervention, not just the average sensing load. This favors explicit energy states: sensing, inference, communication, actuation and recovery. For wireless-powered implants, loss of coupling during therapy should drive a defined safe state. For harvested-energy systems, the controller should know the state of charge and defer non-essential computation when the therapeutic reserve is low.

Closed-loop evidence is broader than one epilepsy implant. A wireless battery-less implant combined neurorecording, compressed deep learning, communication and programmable pharmacological seizure suppression in freely behaving animals [15]. A cortex-adhesive sensor enabled detection-triggered focused-ultrasound seizure control in awake rodents [72]. Stretchable implanted electronics demonstrated ECG sensing with automatically triggered cardiac stimulation and retained function after 14 days in vivo, although power and acquisition remained externally wired [73]. Strain-insensitive adhesive implantable bioelectronics have also coupled physicochemical monitoring to autonomous neuromodulation for glucose regulation in diabetic rats [74]. These systems span electrical, acoustic, pharmacological and neuromodulatory loops. Closed-loop reports should quantify sensing-to-actuation delay, trigger threshold, false-trigger handling, actuator limits, therapeutic reserve, power-loss behavior and in vivo duration; otherwise, evidence supports architectural feasibility rather than chronic therapeutic safety.

## 8. Long-Term Reliability, Biointegration and Safety

### 8.1. Reliability Is Multi-Layered

Long-term reliability in bioelectronic medicine is not one number. It includes material durability, hermeticity or barrier performance, adhesion, electrode electrochemistry, mechanical fatigue, calibration stability, power-source aging, communication availability and software/model behavior. A recent perspective emphasizes that these failure modes interact: a small crack in encapsulation can cause corrosion, which changes electrode impedance, shifts the front-end operating point, and may then be misread by an algorithm as a physiological event [75]. Flexible implant encapsulation therefore needs biocompatibility, low water permeation and mechanical robustness at the same time [76]. Thin-film lifetime engineering further shows that accelerated aging is useful only when the mechanical and environmental stresses represent the intended implant state [77].

### 8.2. Biofouling, Foreign-Body Response and Interface Drift

Implanted chemical sensors face an additional biological failure mode. Protein adsorption, cell adhesion and fibrosis alter analyte transport and interfacial impedance even when the electronics remain intact. Anti-biofouling strategies include hydrophilic and zwitterionic surfaces, biomimetic coatings, drug-eluting layers and active cleaning approaches [78]. For an intelligent system, biofouling is especially problematic because it creates a slowly changing measurement model. An edge algorithm trained on a fresh sensor may compensate some baseline drift, but it cannot recover lost analyte transport or receptor activity without an independent reference. Calibration algorithms should therefore detect progressive loss of sensitivity rather than silently normalize it away.

Insertable biosensors provide an instructive compromise between wearables and fully integrated implants. The sensing material resides below the skin while power, computation and communication can remain in a replaceable wearable monitor [79]. This architecture can reduce the chronic electronics burden but makes wireless interrogation, sensor alignment and long-term transduction stability primary system variables. It is also a useful model for modularity: the components with the shortest technological or battery lifetime do not have to share the same replacement interval as the implanted sensing interface.

Wearable reliability has a different, but equally demanding, exposure profile. Sweat changes ionic strength and pH at the interface; repeated peeling changes adhesive mechanics and electrode contact; textiles and patches experience torsion, abrasion, drying and sometimes washing. Stability should therefore be reported as retention of calibration, baseline and noise after a defined sequence of environmental and mechanical exposures, not only as retention of conductivity. Human-use testing should record placement and remounting because a sensor that performs well when carefully positioned by an investigator may show much larger variance when applied repeatedly by a user.

For implants, sterilization and accelerated aging need to be connected to the actual materials stack. Temperature, radiation or chemical sterilants can change polymer modulus, adhesive strength, receptor activity and thin-film interfaces. Similarly, accelerated soaking at elevated temperature is informative only if the dominant failure mechanism remains water ingress, hydrolysis or corrosion rather than a new degradation pathway created by the test. The most useful lifetime studies combine barrier measurements with functional endpoints such as electrode impedance, calibration slope, leakage current and RF detuning [75,76,77,78,79].

Figure 7 presents reported stability and repeatability evidence from two implemented wearable systems. Liu et al. reported 89.11% retention of the initial glucose-sensor response after more than 10 days, 71.39% after 40 consecutive measurements, and more than 70% of initial electroosmotic-pump flow after more than three weeks [30]. Yang et al. reported 17-day in vivo microneedle monitoring and a 6.9% coefficient of variation in a 100-cycle TPU fatigue test [14]. Because these endpoints are heterogeneous, they are presented in their native form rather than collapsed into a synthetic lifetime score.

## 9. AI Lifecycle, Cyber–Physical Safety and Reporting

Machine learning-enabled medical devices add a lifecycle that conventional sensor papers often omit. International transparency principles for ML-enabled medical devices emphasize intended use, data characteristics, model limitations, performance communication and monitoring after deployment [80]. Good Machine Learning Practice likewise requires representative data, separation of training and test sets, clinically relevant testing, and the monitoring of deployed models [81]. For an intelligent biosensor, these software requirements are inseparable from hardware because the input distribution changes when electrodes age, a patient moves differently, a firmware update changes preprocessing or a sensor batch shifts its baseline.

Security and privacy also couple back to energy. Encrypting every raw high-rate channel and transmitting it continuously is costly; transmitting only a decision can protect privacy but may make an error unauditable. A practical architecture stores short local raw-data windows around detected events, transmits compact features during normal operation, and escalates to richer data when uncertainty increases. The correct balance depends on clinical risk. Closed-loop therapy requires a stronger audit trail and a more conservative update policy than wellness monitoring.

Software updates should be treated as controlled changes to the measurement system. A new filtering routine can alter features presented to the model; a new model can change alert frequency and therefore radio duty cycle; a different decision threshold can alter therapeutic energy demand. Versioning should therefore link firmware, preprocessing, model weights, calibration state and hardware revision. Post-deployment monitoring should look for both statistical model drift and physical sensor drift, because the corrective action is different. Retraining is appropriate for a changing population distribution; it is not a remedy for a delaminating electrode or a failing encapsulation. The transparency and Good Machine Learning Practice principles in Refs. [80,81] support this lifecycle view.

Table 5 provides a minimum reporting checklist. It is intentionally broader than a conventional biosensor table because the system can fail even when analytical sensitivity remains unchanged.

## 10. Cross-Layer Design Rules and Research Priorities

First, choose the transduction mode before optimizing the nanomaterial. The desirable defect density, oxidation state, conductivity and surface chemistry of graphene are different for an amperometric electrode, FET channel, plasmonic layer and resonant RF element. Material optimization without a defined readout often produces record values that cannot survive integration.

Second, optimize regulated energy rather than generator output. A 100 V triboelectric pulse is not equivalent to 100 V from a low-impedance supply. Energy-harvesting papers intended for intelligent wearables should report the energy that reaches storage or the regulated rail under physiological excitation, together with the duty cycle that the stored energy can support.

Third, treat radio activity as an energy event. For many wearable nodes, the largest avoidable load is continuous data transmission. A modest edge model can therefore save energy even if its arithmetic is not extremely efficient, provided that it reduces radio duty cycle. Conversely, an oversized model that requires high-rate acquisition and frequent memory access can consume more energy than it saves.

Fourth, use multimodal sensing only when the channels resolve a specific ambiguity. Adding temperature, pH or motion channels is valuable when they correct a known confounder in the primary biosensor. A multimodal array that merely increases feature count without component controls creates a larger calibration and drift problem.

Fifth, make uncertainty actionable. An uncertainty estimate has little value if the device continues to deliver a therapy or a definitive classification. The system should define what happens when confidence falls: repeat the measurement, acquire a higher-quality channel, transmit raw data, request user action, or enter a safe state.

Sixth, design lifetime experiments around failure physics. Wearable fatigue tests should reproduce strain amplitude, sweat exposure and adhesion cycles. Implant aging should consider water ingress, corrosion, tissue micromotion and encapsulation stress. Accelerated aging is useful only when the acceleration mechanism is related to the real failure pathway.

Seventh, separate model adaptation from sensor calibration. Personalization can improve physiological inference, but it should not be allowed to hide a degrading sensor. A robust device maintains independent indicators of interface quality, electrode impedance or calibration reference so that physiological adaptation and hardware failure remain distinguishable.

Finally, make the replaceable and non-replaceable parts explicit. Wearables can place the battery, radio and processor in a reusable module while using a disposable biochemical interface. Insertable systems can keep the reader outside the body. Implants may separate a long-lived passive sensor from an external powered interrogator. Modularity is not only a manufacturing choice; it can reduce surgical burden and allow faster electronics upgrades without replacing the biological interface.

## 11. Conclusions

Wearable and implantable biosensors should be judged as coupled measurement systems rather than isolated high-performance materials. Component sensitivity or generator peak output is insufficient without matrix-specific calibration, mechanical testing, regulated-energy accounting and telemetry constraints. Edge intelligence is useful when memory, latency, energy and failure behavior are quantified against the communication it replaces. Closed-loop therapy raises the validation threshold because uncertainty, false triggers, power loss and actuator reserve become safety variables. Long-term translation depends on retaining calibrated information under biofouling, encapsulation, fatigue, sterilization and software change. Future reports should pair performance claims with explicit evidence level, lifetime conditions, system-level energy and validation metrics.

Energy autonomy also needs a systems definition. Harvesters, biofuel cells and WPT should be compared after power conversion and storage, with the sensing, computation, radio and actuation duty cycle included. Edge AI can reduce communication energy and enable rapid personalized decisions, but it introduces uncertainty, drift and model-lifecycle requirements. Neuromorphic and in-memory hardware are promising because they reduce data movement provided that device variability is included in validation rather than treated as an implementation detail.

The decisive challenge is long-term reliability. Wearables must remain calibrated through motion, sweat and repeated attachment; implants must survive biofouling, foreign-body response, water ingress, micromotion and limited access for maintenance. Closed-loop systems add the requirement that a sensor failure must not become an unsafe therapeutic action. The most convincing future devices will therefore report a chain of evidence from material identity and interface stability to regulated energy, validated edge inference, wireless safety, biological function and longitudinal performance. That chain—not a record sensitivity, voltage or accuracy value in isolation—is what converts an intelligent biosensor into a credible biomedical system.

## Figures and Tables

**Figure 1:**
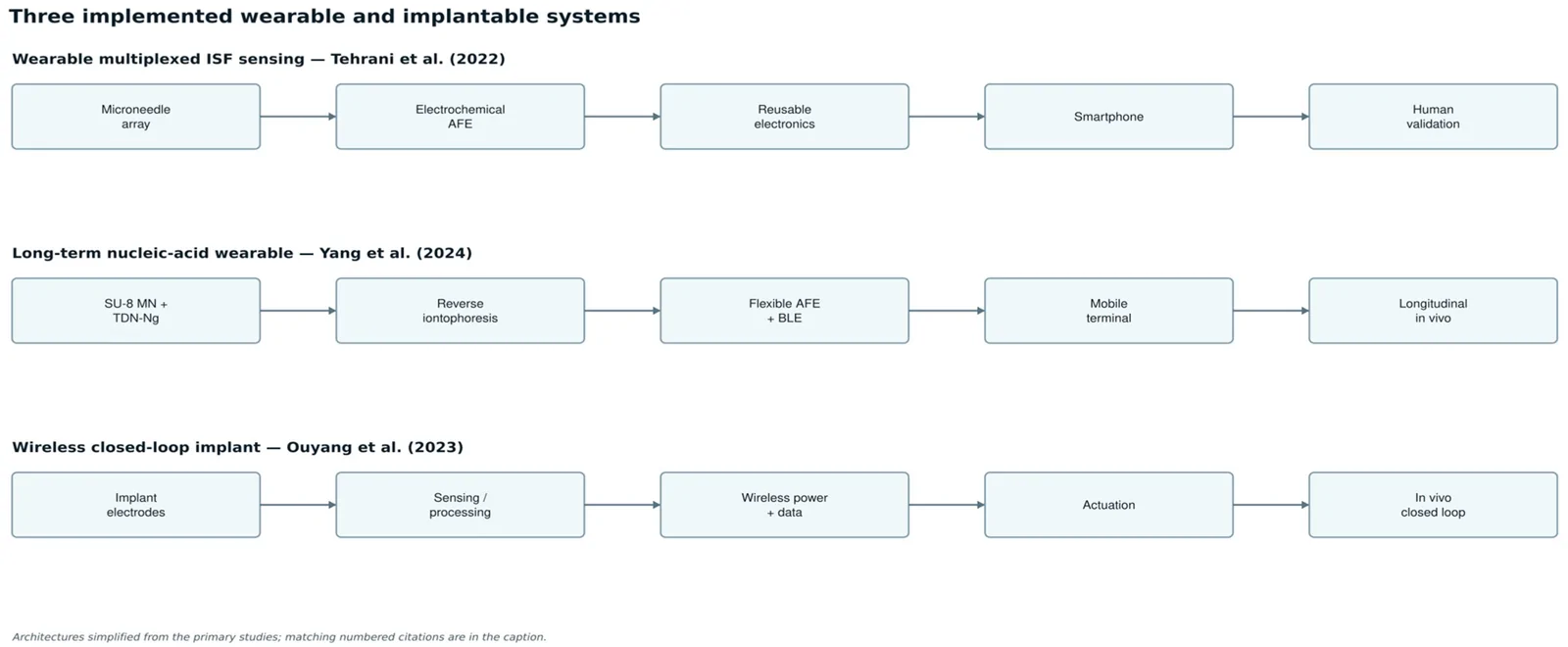
Reference-derived comparison of experimentally implemented wearable and implantable systems. The simplified architectures summarize the implemented signal and energy paths reported for a multiplexed microneedle ISF sensor [13], a longitudinal nucleic-acid wearable with reverse iontophoresis and BLE telemetry [14], and a wireless closed-loop neuromodulation implant [15]. No source artwork is reproduced; the diagram was redrawn from the primary reports.

**Figure 2:**
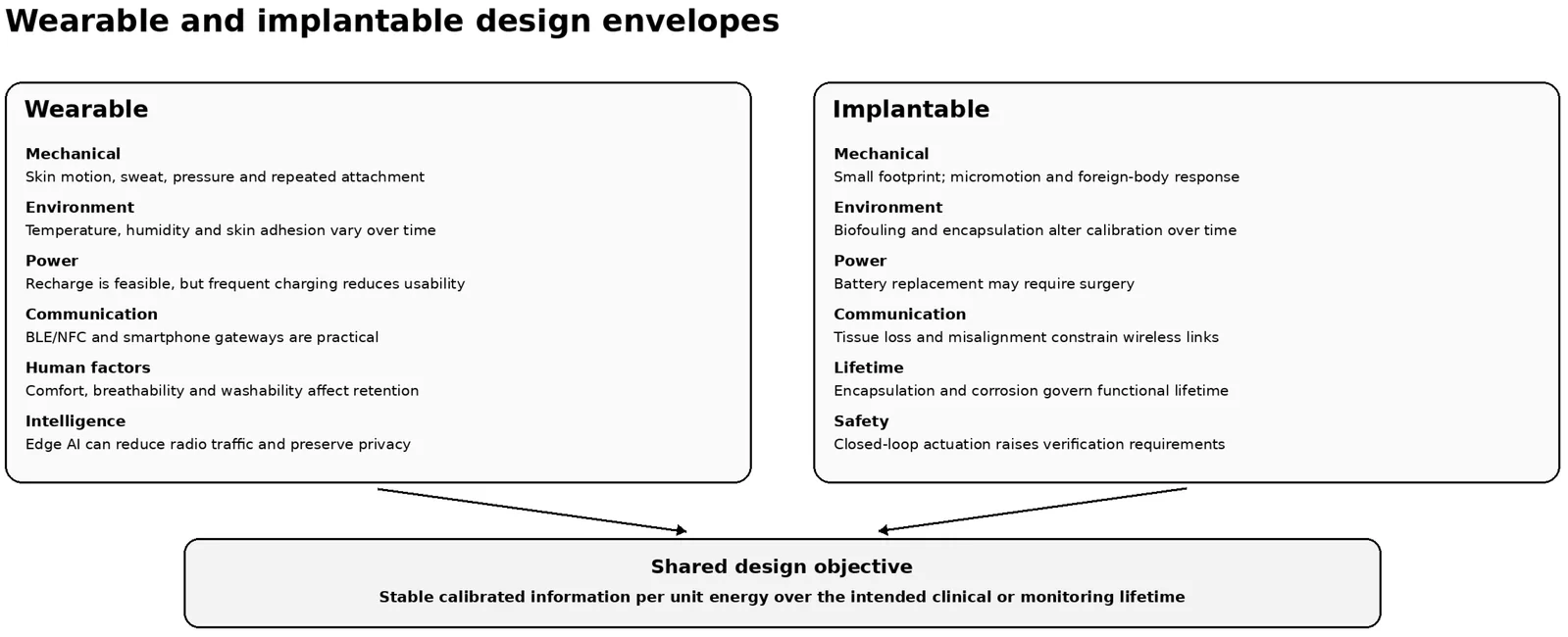
Wearable and implantable design envelopes. The dominant failure modes differ, but both converge on stable information per unit energy over the intended clinical or monitoring lifetime. Original schematic.

**Figure 3:**
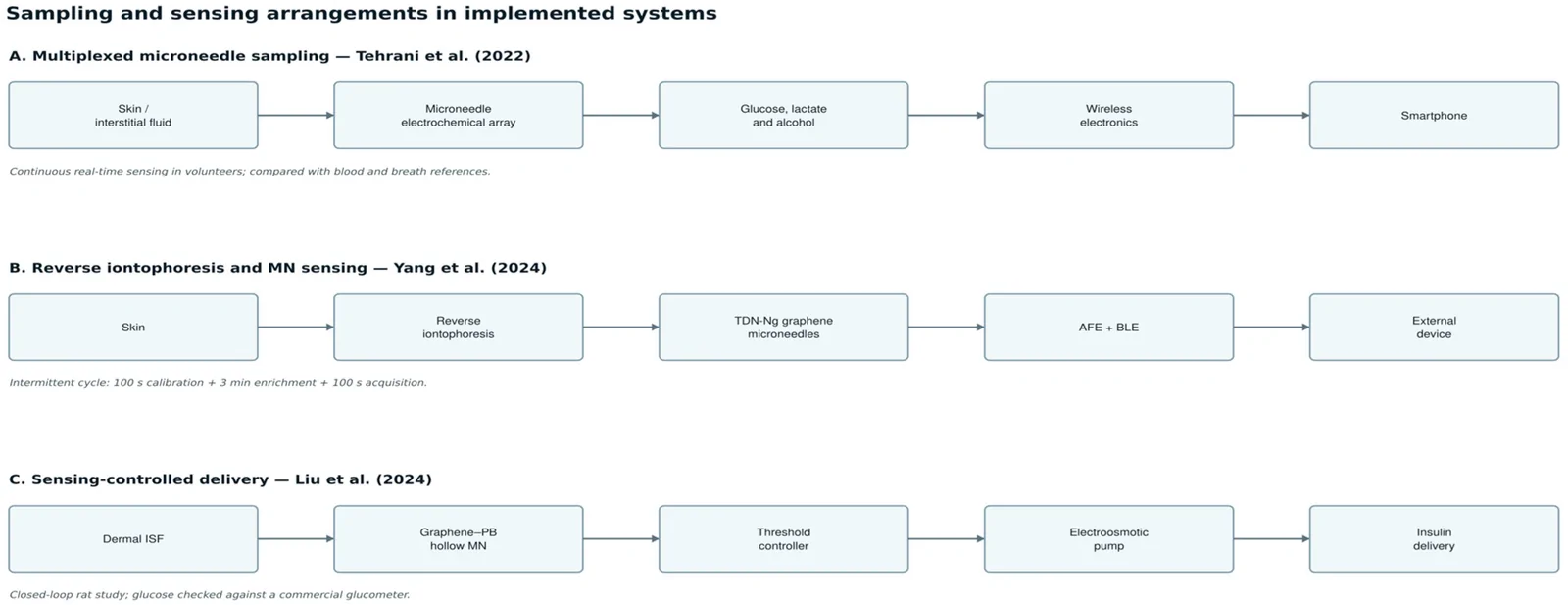
Reference-derived sampling and system arrangements from experimentally implemented platforms. (**A**) Multiplexed microneedle sensing in interstitial fluid with wireless readout and human validation [13]. (**B**) Reverse-iontophoretic enrichment followed by TDN-Ng/graphene microneedle sensing, AFE processing and BLE telemetry [14]. (**C**) Graphene–Prussian-blue hollow-microneedle glucose sensing coupled to threshold-controlled electroosmotic insulin delivery [30]. Architectures were redrawn from the cited primary studies; source artwork is not reproduced.

**Figure 4:**
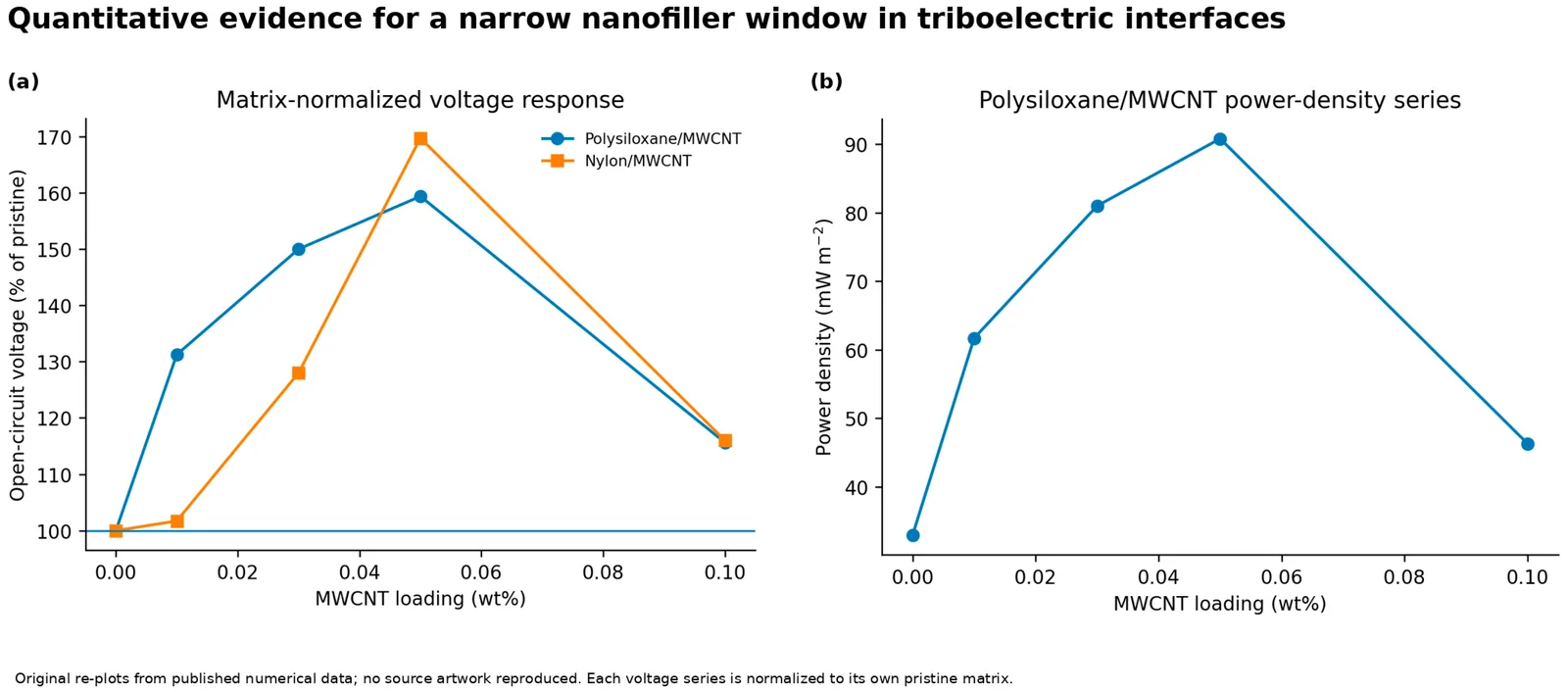
Quantitative evidence for a narrow MWCNT loading window in triboelectric nanocomposites. (**a**) Open-circuit voltage normalized to the pristine polymer for polysiloxane/MWCNT and nylon/MWCNT series. (**b**) Full reported polysiloxane/MWCNT power-density series. Original re-plots from Refs. [41,43] and Ref. [41], respectively; source artwork is not reproduced.

**Figure 5:**
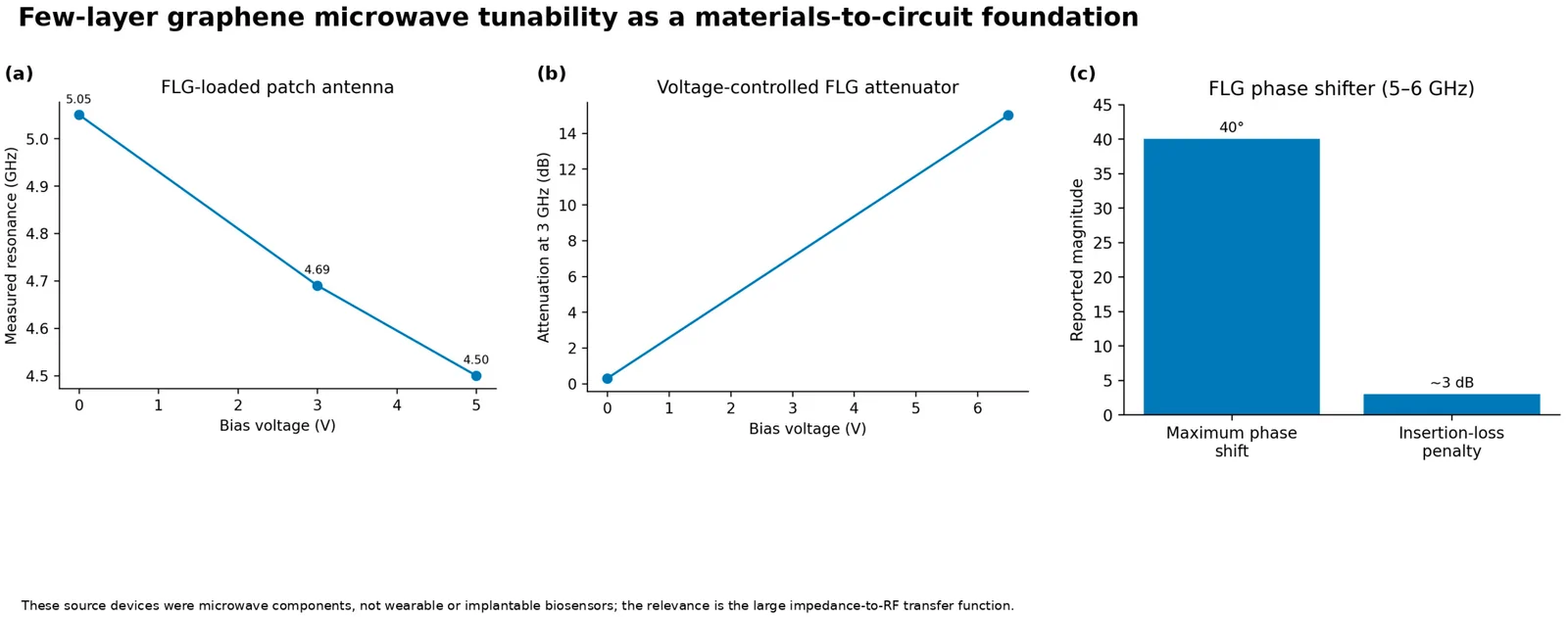
Few-layer graphene microwave tunability as a materials-to-circuit foundation. (**a**) Measured resonance shift in a voltage-controlled FLG patch antenna. (**b**) Representative attenuation change in a tunable FLG microstrip attenuator at 3 GHz. (**c**) Reported maximum phase shift and insertion-loss penalty of an FLG phase shifter. Original re-plots/summaries from Refs. [53,54,55]; the source devices were microwave components, not biomedical sensors.

**Figure 6:**
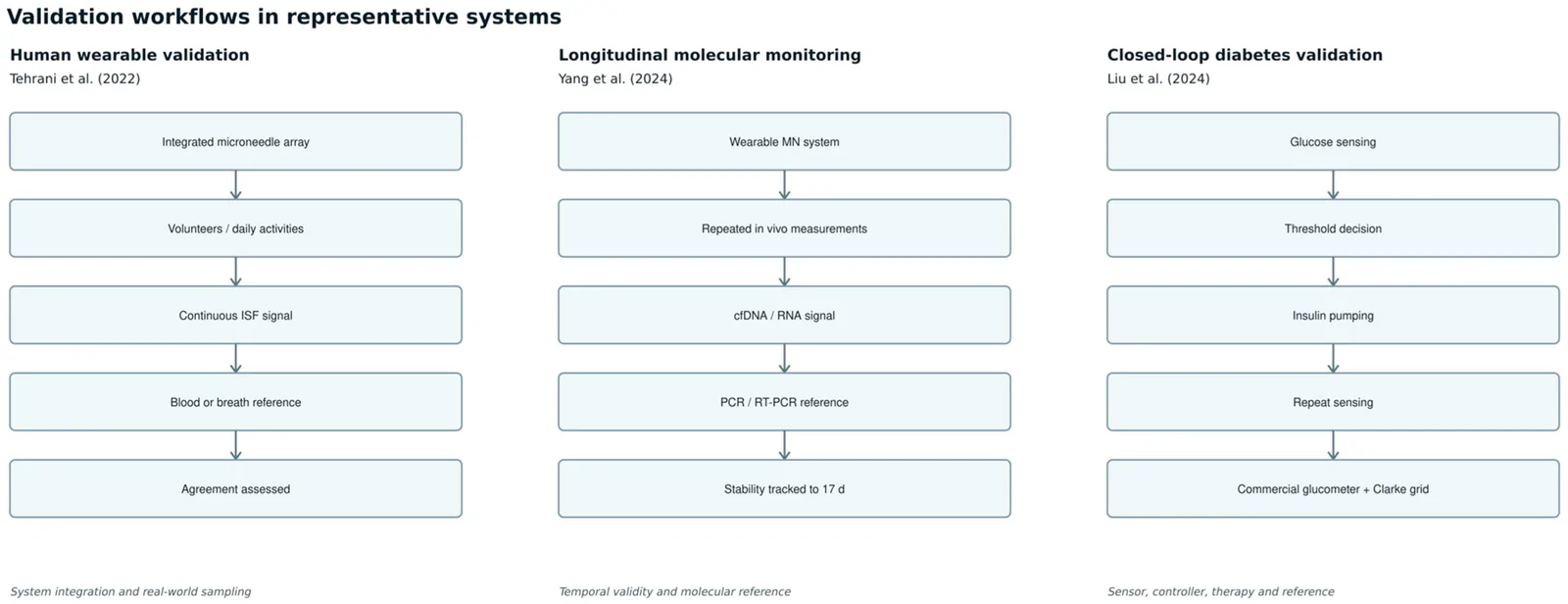
Reference-derived validation workflows for representative wearable and closed-loop systems. The figure summarizes human multi-analyte microneedle validation [13], longitudinal nucleic-acid monitoring with molecular reference methods [14], and sensing-controlled diabetes management with independent glucose reference measurements [30]. The workflows were redrawn from the cited primary studies; source artwork is not reproduced.

**Figure 7:**
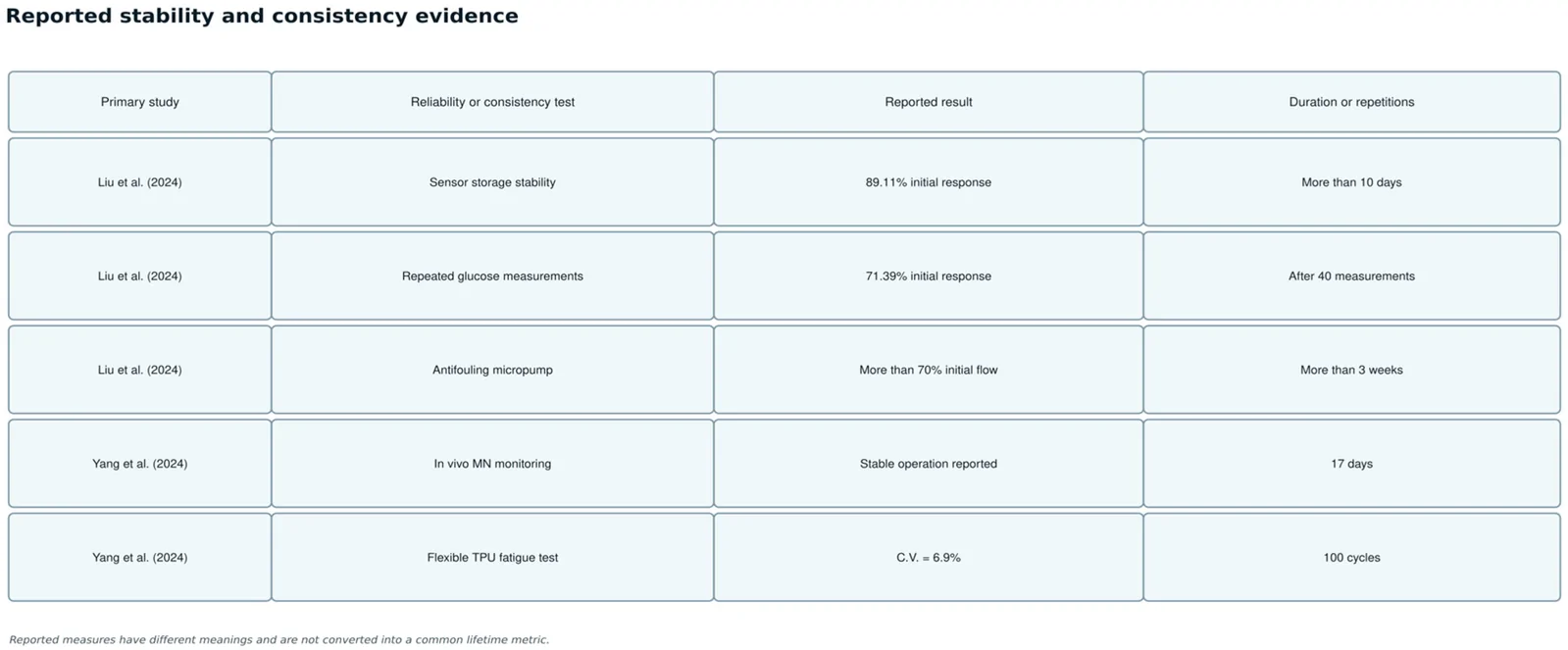
Quantitative stability and consistency evidence from implemented wearable systems. Reported sensor-response retention, repeated-measurement performance and pump-flow retention are from Liu et al. [30]; longitudinal in vivo operation and flexible-substrate fatigue data are from Yang et al. [14]. Values are reproduced as reported in the primary studies and are not normalized across platforms. Source artwork is not reproduced.

**Table 1:** System-level constraints for wearable and implantable intelligent biosensors.

Design Variable	Wearable Systems	Implantable/Insertable Systems	Implication for Co-Design
Mechanical interface	Skin motion, sweat, pressure, repeated attachment	Soft tissue motion, micromotion, foreign-body response	Signal quality and calibration cannot be separated from mechanics
Power access	Rechargeable battery, body energy, near-field power	Strict volume/thermal limits; WPT or harvested physiological energy	Use average regulated power, not generator peak output
Communication	BLE, NFC, smartphone gateways, backscatter	Inductive, RF, acoustic, magnetoelectric or intrabody links	Link budget, alignment and tissue absorption become safety variables
Computation	MCU/TinyML feasible; thermal limits moderate	Ultra-low-power edge processing and event-triggered operation preferred	Computation must justify its energy and verification cost
Environment	Humidity, sweat chemistry, abrasion, washing	Biofluids, corrosion, fibrosis, biofouling, sterilization	Calibration drift and lifetime need environment-specific tests
Failure consequence	Usually loss of data or user adherence	May require surgery or cause unsafe therapy	Fail-safe state and replacement interval are part of performance
Validation	On-body testing and longitudinal wear	Bench + accelerated aging + biocompatibility + in vivo evidence	Application claim should match highest validated level

**Table 2:** Energy-autonomy strategies for wearable and implantable biosensors.

Energy Route	Useful Source/Operating Domain	Strength	Dominant Limitation	System Metric That Should Be Reported
Triboelectric nanogenerator	Motion, contact, respiration, gait	High voltage; sensor and harvester can share interface	Intermittent current; humidity/contact dependence	Energy per event after rectification; calibrated sensing transfer function
Piezoelectric nanogenerator	Strain, vibration, heartbeat, ultrasound	Direct electromechanical conversion; implantable options	Mechanical coupling and fatigue	Power at physiological excitation; strain and cycle history
Enzymatic biofuel cell	Glucose/lactate and other biofuels	Can combine analyte sensing with chemical power generation	Enzyme lifetime and limited power density	Power density over time; fuel concentration; enzyme retention
Thermoelectric/photovoltaic	Body heat gradient/ambient light	Potentially continuous environmental source	Small gradients or variable illumination	Net regulated power under realistic boundary conditions
Wireless power transfer	External RF, magnetic, acoustic or optical source	Controllable and rechargeable without surgery	Alignment, tissue loss/heating, receiver size	Delivered dc power, efficiency, distance, SAR/temperature
Battery/supercapacitor hybrid	Stored energy supporting peak loads	Predictable power for radio and actuation	Finite lifetime, volume, replacement burden	Usable capacity after aging; pulse-power capability; replacement interval

**Table 3:** (**A**). Representative system-level energy evidence. Raw generator metrics are separated from energy delivered after conditioning; NR = not reported. (**B**) Representative sensing, energy, intelligence and telemetry architectures. (**C**) Normalized comparison of representative integrated wearable/implantable systems. NR = not reported; E1–E4 are defined in Section 2.

**(A) Representative System-Level Energy Evidence. Raw Generator Metrics Are Separated from Energy Delivered After Conditioning; NR = Not Reported.**						
**System/Ref.**		**Source**	**Raw Output**	**Conditioning/Storage**	**Usable Evidence**	**Interpretation**
Polysiloxane/MWCNT TENG [41]		Mechanical	32–51 V; 33.0–90.8 mW m^−2^	Complete regulated node NR	NR	E1 harvester, not autonomous biosensor
GO/silk-fibroin/PCL TENG [47]		Mechanical	Generator output reported	Biomedical-compatible fibrous architecture	Regulated rail NR	E1–E2 enabling evidence
Solar wearable platform [58]		Photovoltaic	PV input	Cold start + MPPT + regulation	Stable 3.3 V; four sensor nodes	E2–E3 system autonomy
Battery-less impedance implant [59]		Wireless power	External RF	On-chip regulation + EIS	1.8 V ASIC; 274 μA	E3 powered implant
Wireless closed-loop implant [15]		Wireless power	External field	On-board power management	Recording + communication + actuation	E3 integrated closed loop
**(B) Representative sensing, energy, intelligence and telemetry architectures**						
**Example**		**Interface/transduction**	**Power/intelligence**	**Telemetry/output**	**Evidence level and main limitation**	
Wearable sweat glucose + ML [31]		Electrochemical sweat sensing	Battery-powered wearable; ML analysis	Smartphone-linked	On-body; performance depends on sweat dynamics and calibration	
Wearable non-enzymatic sweat interface [32]		Multichannel electrochemical features	ML prediction of amino acids and pH	Wireless microsystem	Human exercise testing; model/generalization burden	
Bipolar self-powered miRNA system [33]		Enzyme-catalyzed self-powered sensing	Self-powered signal + ML	Commercial chip/smartphone	Integrated biochemical prototype; clinical cohort validation still needed	
Wireless graphene magnetoelastic biosensor [28]		Resonant magnetoelastic interface	Passive/remote interrogation	Wireless resonance	Biosensing demonstrated; packaging and longitudinal biointerface remain important	
3D-printed microneedle glucose sensor [29]		Electrochemical microneedle	Integrated electronics	Wireless glucose monitoring	Wearable architecture; microneedle fouling and insertion repeatability matter	
FLG tunable antenna/phase/attenuation [52,53,54,55,56]		Voltage-dependent graphene impedance	Externally biased circuit component	RF amplitude, phase or resonance	Materials-to-circuit foundation; not direct biomedical evidence	
In-body/on-body IBC [57]		Body channel as communication medium	Low-power communication architecture	Intrabody link	Biomedical channel characterization; application-specific coupling required	
**(C) Normalized comparison of representative integrated wearable/implantable systems. NR = not reported; E1–E4 are defined in Section 2.**						
**System/Ref.**	**Target/transduction**	**Matrix/setting**	**Performance**	**Drift/mechanical**	**Power/telemetry**	**Evidence**
Integrated MN array [13]	Glucose+lactate/alcohol; electrochemical	Human ISF	Correlated with blood/breath reference	On-body multiplexed	Reusable electronics; smartphone	E3
Vertical-graphene MN [34]	Ketone/glucose; electrochemical	Human ISF	234.18 μA mm^−1^ cm^−2^; LOD 1.21 μM	MN mechanics; drift NR	Integrated wearable	E3
Smartphone MN-CGM [35]	Glucose; electrochemical	Human ISF	14 days; MARD 10.2%	14-day retention	Smartphone controlled	E4-like
Wireless sweat CRP [36]	CRP; immunosensing	Human sweat	Real-time monitoring	On-body; long-term recalibration NR	Wireless patch	E3
Wireless PEC MN [37]	ISF chemistry; PEC	Wearable ISF	Zero-bias longitudinal architecture	Stability-oriented design	Wireless electronics	E2–E3
Battery-less implant [59]	Impedance	Animal implant	1% impedance precision	In vivo test	Wireless powered	E3
Closed-loop MN [60]	Glucose → insulin	Diabetic rats	0.212 μA/mm; LOD 14 μm; MARD 9.96%	In vivo closed loop	PCB + ultrasonic pump	E3

**Table 4:** (**A**) Edge AI and neuromorphic strategies for wearable and implantable biosensors. (**B**) Quantitative edge AI benchmarks relevant to wearable biomedical deployment.

**(A) Edge AI and Neuromorphic Strategies for Wearable and Implantable Biosensors.**					
**Strategy**		**Primary benefit**	**Hardware consequence**	**Validation requirement**	
Feature-first classical ML		Low memory and interpretable inputs	Simple MCU/DSP; low radio traffic	Features must remain stable across sensor drift and users	
Quantized TinyML		Compact deep inference	Reduced memory and arithmetic precision	Accuracy after quantization; energy/inference; worst-case latency	
Pruning/knowledge distillation		Smaller network at similar task accuracy	Fewer operations and memory accesses	Re-evaluate minority classes and edge cases after compression	
Event-driven/spiking neural network		Compute only when events occur	Asynchronous or neuromorphic hardware	Event encoding stability; false event rate; timing tolerance	
In-memory/memristive computing		Reduces data movement	Analog variability and endurance become model parameters	Device-to-device variation, retention, write endurance, temperature drift	
On-device personalization		Adapts to subject-specific baseline	Additional memory, update energy and update logic	Guard rails, version control, rollback, independent validation	
Uncertainty/OOD detection		Prevents confident decisions outside training domain	Extra computation but safer exception handling	Prospective thresholds and failure-state testing	
**(B) Quantitative edge AI benchmarks relevant to wearable biomedical deployment.**					
**System/Ref.**	**Task**	**Model/memory**	**Latency**	**Energy/power**	**Validation/OOD**
EdgeECG [67]	5-class arrhythmia; MIT-BIH	2680 parameters; STM32F103, 64 kB SRAM	25 ms	0.156 mJ/inference	98.07% overall; 80.73% patient-independent; OOD NR
TinyML ECG [68]	ECG anomaly detection	Pruned + quantized embedded model	Real-time	0.024 mW reported	92.3%; external/OOD NR
Printed neuromorphic wearable [10]	Multimodal sensing	Chip-less printed local processing	Event/local	Reduced off-body compute	On-body proof-of-concept; longitudinal OOD NR

**Table 5:** (**A**) Quantitative lifetime and reliability evidence relevant to long-term wearable/implantable operation. (**B**) Minimum reporting set for intelligent energy-autonomous wearable and implantable biosensors.

**(A) Quantitative Lifetime and Reliability Evidence Relevant to Long-Term Wearable/Implantable Operation.**				
**Example/Ref.**	**Stress/Duration**	**Functional Endpoint**	**Implication**	**Evidence**
Smartphone MN-CGM [35]	14 days, human ISF	MARD 10.2%	Multi-day calibration retention	E4-like
Stretchable implant [73]	14 days in vivo mouse	ECG + stimulation retained	Mechanical stability; longer biocompatibility needed	E3
Drug-delivery implant [82]	Up to 8 weeks preclinical	Insulin transport through fibrous capsule	Actuation mitigates encapsulation-related loss	E3 longitudinal
Encapsulation monitor [83]	In vitro + in-tissue	Frequency shift tracks water ingress	Direct barrier-lifetime observable	E1–E3 method
Anti-biofouling [78]	Chronic sensor literature	Drift, sensitivity, MARD, lifetime	Biofouling couples to calibration loss	Review synthesis
Insertable sensors [84]	Long-term subdermal use	Migration, coupling, fibrosis	Reader alignment/tissue response are system variables	Translation synthesis
**(B) Minimum reporting set for intelligent energy-autonomous wearable and implantable biosensors.**				
**Domain**	**Minimum information**		**Reason**	
Material/interface	Material identity, batch, functionalization, thickness, roughness, receptor chemistry, hydrated mechanics		Defines the actual biological and electrical interface	
Transduction	Calibration curve, selectivity, noise, response time, hysteresis, drift, impedance/spectral context		Separates analytical performance from a single peak signal	
Power	Average and peak load; harvester/WPT input; rectifier/regulator efficiency; storage capacity and aging		Demonstrates that the complete node, not just the harvester, is autonomous	
Front end	Sampling rate, bandwidth, input-referred noise, ADC resolution, excitation amplitude		Allows reconstruction of the measurement chain	
Edge AI	Dataset units (devices/subjects), split strategy, model size, latency, energy/inference, uncertainty/OOD rule		Prevents data leakage and unqualified accuracy claims	
Wireless	Protocol, frequency, range/depth, data rate, duty cycle, power, alignment, tissue heating/SAR where relevant		Makes telemetry and safety comparable	
Mechanical/environmental	Bending/stretching cycles, adhesion, sweat/PBS aging, temperature/humidity, sterilization		Links device lifetime to the intended use environment	
Biological	Matrix, cell/tissue model, in vivo duration, inflammatory/fouling assessment		Defines the level of biomedical evidence	
Closed-loop safety	Trigger logic, latency, fail-safe state, actuator limits, power-loss behavior		Required when sensing can initiate therapy	
Software lifecycle	Version, update policy, recalibration method, post-deployment monitoring		Model behavior can change after the hardware is deployed	

## Data Availability

No new primary experimental data were generated in this Review. Quantitative values in Figure 4, Figure 5 and Figure 7 were re-plotted or summarized from the published sources cited in the corresponding captions. No source artwork was reproduced.
